# ERCC1 expression status predicts the response and survival of patients with metastatic or recurrent cervical cancer treated via platinum-based chemotherapy

**DOI:** 10.1097/MD.0000000000009402

**Published:** 2017-12-22

**Authors:** Hyewon Ryu, Ik-Chan Song, Yoon-Seok Choi, Hwan-Jung Yun, Deog-Yeon Jo, Jin Man Kim, Young Bok Ko, Hyo Jin Lee

**Affiliations:** a Department of Internal Medicine; b Department of Pathology; c Infection Control Convergence Research Center; d Department of Obstetrics and Gynecology, Chungnam National University College of Medicine, Daejeon, South Korea.

**Keywords:** chemotherapy, cisplatin, ERCC1, uterine cervical carcinoma

## Abstract

The deoxyribonucleic acid (DNA) repair gene encoding the excision-repair cross-complementation group 1 (ERCC1) protein is known to predict the response to platinum-based chemotherapy. Our aim was to explore whether ERCC1 expression predicted tumor response and survival in patients with recurrent or metastatic cervical cancer treated via platinum-based chemotherapy. We analyzed 32 such patients. ERCC1 expression was assessed immunohistochemically in pretreatment biopsy samples. Of the 32 patients, 13 (40.6%) were ERCC1 high. ERCC1-low patients exhibited a significantly higher response rate (73.7%) than did others (15.4%). The median progression-free survival differed significantly by ERCC1 status, being 135 days in ERCC1-high and 242 days in ERCC1-low patients (hazard ratio, 2.428; 95% confidence interval, 1.145–5.148, *P* = .032). Overall survival was significantly longer in ERCC1-low (617 days) than in ERCC1-high (320 days) patients (hazard ratio, 2.322; 95% confidence interval, 1.051–5.29; *P* = .037). Thus, pretreatment ERCC1 expression status can be used to predict tumor response and survival of patients with recurrent or metastatic uterine cervical cancer receiving platinum-based chemotherapy.

## 1 Introduction

Cervical cancer is the third most common gynecological cancer in the United States and the most common gynecological cancer worldwide.^[1,2]^ An estimated 528,000 new cases of cervical cancer were diagnosed in 2012, and 266,000 patients died.^[1]^ Cervical cancer can often be treated successfully when detected early. However, patients who exhibit distant metastases at initial presentation or at relapse can rarely be cured. Chemotherapy (usually platinum doublets) remains the standard treatment for such patients.^[3–5]^ However, conventional chemotherapy is neither curative nor associated with long-term disease control.^[6]^ Thus, the identification of factors better predicting treatment response and survival outcome is critical.

Deoxyribonucleic acid (DNA) repair is critically involved in the development of cisplatin resistance.^[7]^ Platinum salts bind to DNA to create platinum–DNA adducts,^[8]^ which then covalently cross-link DNA strands, inhibiting DNA replication. Nucleotide excision/repair plays a central role in adduct removal and is associated with resistance to platinum-based chemotherapy.^[7]^ The excision repair cross-complementation group 1 (ERCC1) protein is a key mediator of cisplatin resistance. It forms the rate-limiting enzyme of the nucleotide excision/repair pathway that removes platinum–DNA adducts.^[9–11]^

In vitro studies have shown that platinum resistance is associated with ERCC1 mRNA expression in ovarian, cervical, testicular, bladder, and non-small-cell lung cancer cell lines.^[12,13]^ Additionally, some clinical studies have revealed that ERCC1 expression is correlated with resistance to platinum-based chemotherapy and poor prognosis in patients with several types of tumor,^[12,14–17]^ suggesting that the DNA-damage repair capacity plays an important role and is involved in resistance to cisplatin-based chemotherapy or radiotherapy. Thus, we explored whether ERCC1 status predicted tumor response and survival in patients with metastatic or recurrent uterine cervical cancer receiving platinum-based chemotherapy.

## 2 Methods

### 2.1 Patients and treatment

Between October 2004 and January 2011, 32 patients with recurrent or metastatic uterine cervical cancer, for whom pretreatment tissue samples were available, were treated with platinum doublets at Chungnam National University Hospital, and their medical records were reviewed retrospectively. We analyzed patient demographics, Eastern Cooperative Oncology Group (ECOG) performance status (PS), the histological type of disease, site of disease, prior use of radiosensitizers, the chemotherapy regimen, hemoglobin level prior to chemotherapy, date of disease progression, and survival status at the last follow-up. The chemotherapy regimens included cisplatin/paclitaxel, carboplatin/paclitaxel, cisplatin/5-fluouracil, and cisplatin/topotecan. Tumor responses were assessed every 2 or 3 cycles using the response evaluation criteria in solid tumors (RECIST) system, version 1.1. All patients gave written informed consent. The study protocol was approved by our institutional review board.

### 2.2 Immunohistochemistry

ERCC1 expression was analyzed by immunohistochemistry (IHC). We prepared paraffin-embedded tissue sections from all cervical cancer samples. Sections (3 μm thick) of the paraffin blocks were subjected to IHC using the mouse EnVision-HRP detection system (Dako, Carpinteria, CA). A monoclonal mouse antibody against ERCC1 (Clone 8F1; Thermo, Fremont, CA) was used for IHC. Sections were placed in 10 mM sodium citrate buffer (pH 6.0) and, after deparaffinization and antigen retrieval in a pressure cooker running at full power for 4 minutes, were exposed to 3% (v/v) hydrogen peroxide for 10 minutes. The primary antibody was diluted 1:800 with a background-reducing diluent (Dako) and incubated overnight at 4°C in a humidified chamber. The slides were then incubated with the EnVision reagent for 30 minutes, followed by incubation with the DAB chromogen for 5 minutes; thereafter, they were counterstained with Meyer hematoxylin and mounted. Careful rinses using several changes of TBS-0.3% (v/v) Tween were performed between each step. A mouse IgG1 isotype sample (lacking the primary antibody) served as the control. Cells exhibiting nuclear staining were considered to be positive.

### 2.3 Evaluation of ERCC1 expression

ERCC1 nuclear expression was assessed semiquantitatively using the immunoreactive scoring (IRS) system. IRS is based on staining intensity (scored on a 0–3 scale, where 0 = no staining, 1 = weak staining, 2 = moderate staining, and 3 = strong staining) and staining extent (the percentage of positive cells, scored on a 0–1 scale, where 0 = no staining, 0.1 = 1%–9%, 0.5 = 10%–49%, and 1 = 50%–100% staining). A final semiquantitative *H* score, ranging from 0 to 3, is obtained by multiplying the scores. The median *H* score served as the cutoff separating ERCC1-high from ERCC1-low tumors.^[18]^

### 2.4 Statistical analysis

Categorical variables were compared using the *χ*^2^ test. Survival probability analyses were performed using the Kaplan–Meier method. Progression-free survival (PFS) was defined as the interval from the first treatment to the date of documented disease progression. Overall survival (OS) was defined as the interval from the first treatment to the date of death from any cause. The significance of between-group differences was assessed using the log-rank test. *P* values <.05 were considered to indicate statistical significance. All statistical analyses were performed with SPSS software (ver. 22.0; SPSS Inc, Chicago, IL).

## 3 Results

### 3.1 ERCC1 expression and clinical features

**Figure 1:**
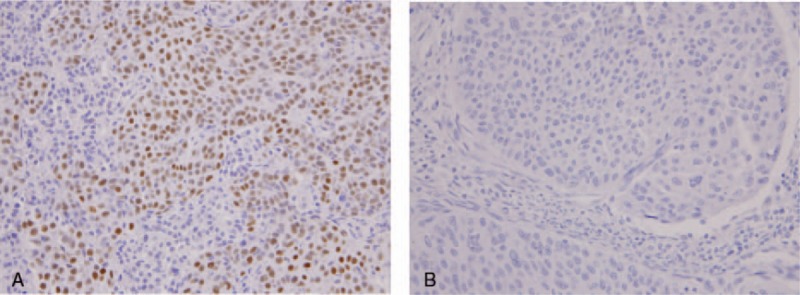
Representative immunohistochemical staining of ERCC1 in uterine cervical carcinoma. Expression of ERCC1 was assessed by immunohistochemistry. Expression of ERCC1 protein (nuclear signal, brown) was detected in the nuclei of cancer cells. ERCC1-high group (A) and ERCC1-low group (B). Original magnification, × 400. ERCC1 = excision repair cross-complementation group 1.

Patient characteristics are shown in Table 1. The median patient age was 51 years (range, 34–67 years). ERCC1 expression was localized to the nucleus (Fig. 1) and the median *H* score was 1.5. Patients were thus divided into ERCC1-low (score ≤ 1.5) and ERCC1-high (score > 1.5) groups. Of the 32 patients, 13 (40.6%) were ERCC1 high and 19 (59.4%) were ERCC1 low. The 2 groups did not differ in terms of age, ECOG PS, histological type, site of disease, disease status, prior radiosensitizer use, chemotherapeutic regimen, or hemoglobin level prior to chemotherapy (Table 1).

**Table 1:**
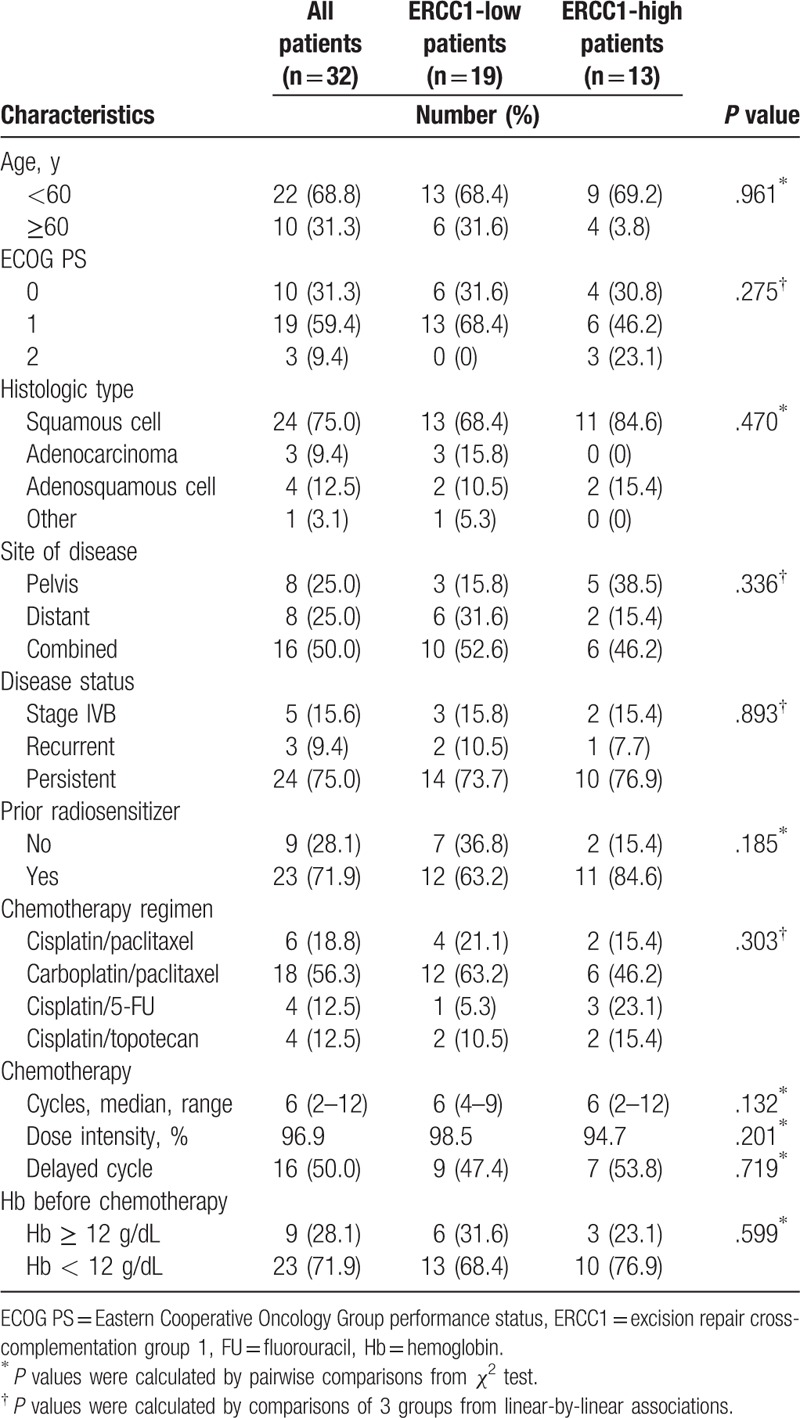
Patient characteristics.

### 3.2 Relationship between ERCC1 expression and treatment response

The overall complete response rate was 12.5% (4 of 32 patients). Twelve patients showed partial response, 10 patients exhibited stable disease, and 6 showed disease progression. ERCC1-low patients exhibited a significantly higher response rate (14/19, 73.7%) and disease control rate (18/19, 94.7%) than did ERCC1-high patients (2/13, 15.4% and 8/13, 61.5%; *P* = .001 and.018, respectively; Table 2). Moreover, ERCC1-low patients had a significantly higher complete response rate (4/19, 21.1%) than did ERCC1-high patients (0/13, 0%; *P* = .001; Table 2).

**Table 2:**
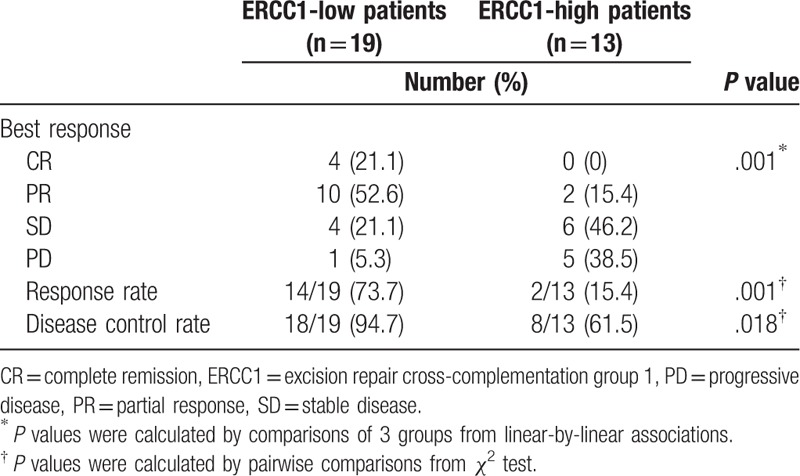
Expression of ERCC1 and response to chemotherapy.

### 3.3 Relationship between ERCC1 expression and survival

**Figure 2:**
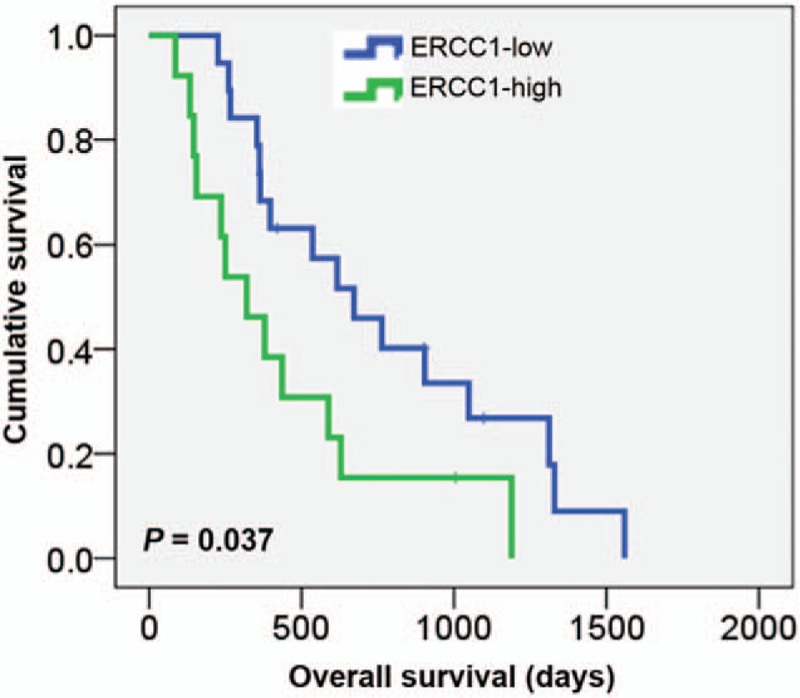
Overall survival according to ERCC1 expression status. ERCC1-high patients had shorter overall survival than ERCC1-low ones. ERCC1 = excision repair cross-complementation group 1.

**Figure 3:**
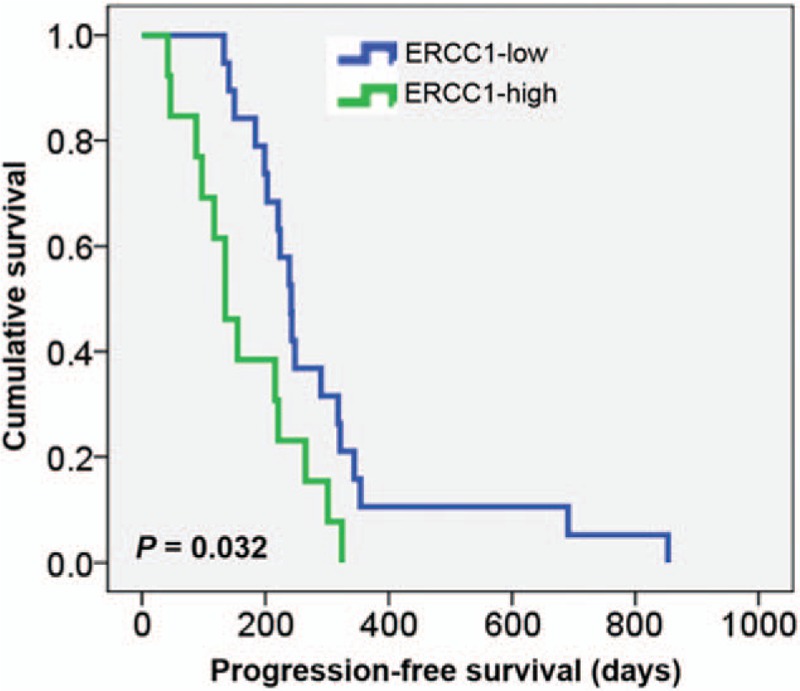
Progression-free survival according to ERCC1 expression status. ERCC1-high patients had worse progression-free survival than ERCC1-low ones. ERCC1 = excision repair cross-complementation group 1.

We compared PFS and OS by ERCC1 expression status. The median follow-up time was 14.3 months (range, 2.9–52.0 months). The median OS of ERCC1-high patients was 320 days and that of ERCC1-low patients was 617 days (hazard ratio, 2.322; 95% confidence interval, 1.051–5.129; *P* = .037; Fig. 2, Table 3). The median PFS was also significantly poorer in ERCC1-high than in ERCC1-low patients (135 vs 242 days; hazard ratio, 2.428; 95% confidence interval, 1.145–5.148; *P* = .032; Fig. 3, Table 3). Univariate and multivariate analyses indicate that high ERCC1 expression was an independent risk factor predicting OS in advanced uterine cervical cancer patients treated with platinum-based chemotherapy.

**Table 3:**
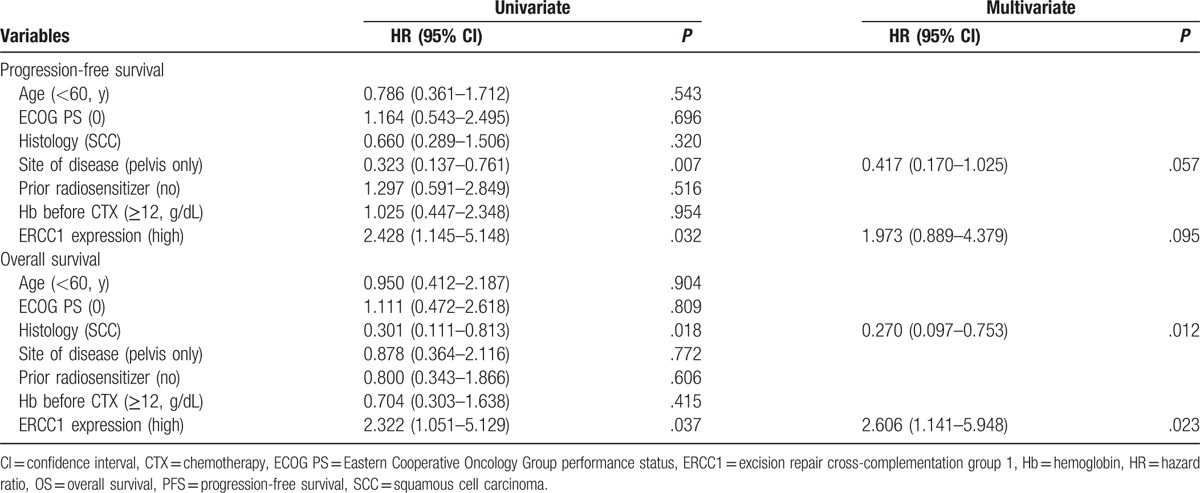
Univariate and multivariate analyses of PFS and OS with ERCC1 expression.

## 4 Discussion

Metastatic or recurrent cervical cancer remains a major cause of female death. Affected patients commonly receive palliative chemotherapy featuring platinum doublets, but the prognosis is extremely poor. The current 5-year survival rate is only 17%. Therefore, biomarkers predicting outcomes after platinum doublet treatment are required for patients with metastatic or recurrent cervical cancer.^[19]^

Effective DNA repair confers cisplatin resistance, and the gene encoding ERCC1 is crucial in this context. Cisplatin–DNA adducts are removed via nucleotide excision repair, and relationships between ERCC1 expression and resistance to platinum compounds have been observed in patients with various cancers, including cervical cancer.^[12,14–16,20,21]^ We hypothesized that ERCC1 would be associated with the response to treatment, and explored whether ERCC1 expression could serve as a biological marker predicting the clinical outcomes of patients with advanced uterine cervical cancer undergoing platinum-based chemotherapy. We found that low ERCC1 expression was associated with a significantly better therapeutic response and longer survival. ERCC1-low patients exhibited a significantly better overall response rate (73.7% vs 15.4%) and improved disease control (94.7% vs 61.5%) than did ERCC1-high patients, consistent with findings from patients with head-and-neck squamous cell, esophageal, and bladder cancers.^[22–24]^ Furthermore, low ERCC1 expression was associated with significantly longer PFS and OS. The PFS of patients with ERCC1-low cancer was 242 days, compared with 135 days for ERCC1-high patients (*P* = .032). The OS of ERCC1-low patients was also better than that of ERCC1-high patients, consistent with a finding from locally advanced cervical cancer patients undergoing cisplatin monotherapy.^[25]^

Several studies have explored whether ERCC1 status is a useful marker of cervical cancer prognosis. Britten et al^[12]^ found that the ERCC1-encoding mRNA level predicted cisplatin resistance in human cervical cancer cell lines. Park et al^[26]^ reported that low-level ERCC1 expression independently predicted prolonged disease-free survival in patients with uterine cervical cancer undergoing cisplatin-based neoadjuvant chemotherapy. Recently, Karageorgopoulou et al^[27]^ reported that ERCC1 expression status was significantly prognostic of survival in patients with metastatic or recurrent cervical cancer undergoing cisplatin-based chemotherapy. In contrast, Doll et al^[28]^ found that low-level ERCC1 expression was associated with poorer survival in patients with cervical cancer receiving radiation alone, suggesting that the poor outcomes of patients with low-level ERCC1 expression were not related directly to the repair of radiation-induced DNA damage by the ERCC1-dependent DNA repair pathway, but rather to the emergence of a more aggressive tumor phenotype reflecting a reduced DNA repair capacity when radiation alone was prescribed.

Our study has certain limitations. First, our work was retrospective in nature and was not confined to data gathered over a short period. Second, our patient sample was small; our findings must be interpreted with caution. A well-designed prospective study with a large patient sample is required. Despite these limitations, we have shown that the pretreatment ERCC1 level in tumor cells was related inversely to the outcomes of platinum-based chemotherapy in patients with metastatic or recurrent uterine cervical cancer.

In conclusion, we showed that ERCC1 expression patterns in pretreatment specimens predicted tumor response and survival in patients undergoing platinum-based chemotherapy to treat metastatic or recurrent uterine cervical cancer. Thus, ERCC1 expression status may usefully predict outcomes in such patients.
